# Case study of a rhizosphere microbiome assay on a bamboo rhizome with excessive shoots

**DOI:** 10.48130/FR-2021-0010

**Published:** 2021-06-24

**Authors:** Fuqiang Cui, Yifan Yang, Mengyuan Ye, Wei Wei, Wenqian Huang, Ying Wu, Xi Jiao, Xiaoxue Ye, Shutong Zhou, Zhubing Hu, Yinhai Zhang, Renyi Gui, Wenwu Wu, Kim Yrjälä, Kirk Overmyer, Shenkui Liu

**Affiliations:** 1 State Key Laboratory of Subtropical Silviculture, Zhejiang A&F University, Lin’an 311300, Hangzhou, China; 2 School of Agricultural Engineering, Jiangsu University, Zhenjiang 212013, China; 3 Organismal and Evolutionary Biology Research Program, Faculty of Biological and Environmental Sciences, and the Viikki Plant Science Centre, University of Helsinki, P.O. Box 65 (Viikinkaari 1), FI-00014 Helsinki, Finland; 4 Key Laboratory of Plant Stress Biology, School of Life Sciences, Henan University, Kaifeng 475004, China; 5 Weifang Inspection and Testing Center, Weifang 261100, China

**Keywords:** Bamboo, rhizosphere, microbial diversity, *Burkholderia*, shoot clusters

## Abstract

Young moso bamboo shoots are a popular seasonal food and an important source of income for farmers, with value for cultivation estimated at $30,000 per hectare. Bamboo also has great environmental importance and its unique physiology is of scientific interest. A rare and valuable phenomenon has recently appeared where a large number of adjacent buds within a single moso bamboo rhizome have grown into shoots. Although of practical importance for the production of edible shoots, such occurrences have not been scientifically studied, due to their rarity. Analysis of collected reports from enhanced shoot production events in China showed no evidence that enhanced shoot development was heritable. We report the analysis of the rhizosphere microbiome from a rhizome with 18 shoots, compared to rhizomes having one or no shoots as controls. The community of prokaryotes, but not fungi, correlated with the shoot number. *Burkholderia* was the most abundant genus, which was negatively correlated with rhizome shoot number, while *Clostridia* and *Ktedonobacteria* were positively correlated. Two *Burkholderia* strains were isolated and their plant-growth promoting activity was tested. The isolated *Burkholderia* strains attenuated the growth of bamboo seedlings. These data provide the first study on excessive shoot development in bamboo, which will facilitate hypothesis building for future studies.

## INTRODUCTION

Moso bamboo (*Phyllostachys edulis*) is the most widespread bamboo species in Asia, in China it covers approximately 4 million hectares^[1]^. Mechanisms for its rapid growth have been previously studied^[2]^, and it has been presented as an ideal plant for bioeconomy to meet challenges of sustainability^[3]^. The young shoots of moso bamboo are a very popular seasonal food. Moso bamboo shoots are rich in fiber and nutrients, making them a desirable health food increasing in demand^[4,5]^. Income from the bamboo shoot business has reached $30,000 per hectare in advanced cultivation areas^[6]^. Techniques to improve shoot production rely mainly on fertilization management^[7−12]^. Moso bamboo propagates vegetatively through rhizomes, and each node of a rhizome possesses a single bud. The bud number in one rhizome can be from in the tens to over 100, depending on the rhizome length and environmental conditions. Most buds remain dormant with less than 5% developing into shoots^[13]^. Given the energy demands of the extremely rapid growth in bamboo shoots (> 1 meter per day at maximum), the germination of too many shoots from a rhizome is detrimental^[14]^; energy waste from competition and decay of excess shoots selects against its occurrence. For well-cultivated bamboo farms, where shoots are collected at a very young stage, increased shoot number, however, would be economically desirable and have practical labor-saving advantages. Increased numbers of shoots on a rhizome would improve the visibility of soil mounds pushed up from young shoots, facilitating simple detection and harvest of randomly distributed underground shoots.

For moso bamboo, usually one or two neighboring buds develop into shoots. Four to six neighboring shoots within a rhizome is uncommon. Only very few cases have been reported, with no scientific reports available, where more than ten shoots have developed densely along one rhizome and no research has previously been conducted regarding multiple shoots. Here we term this rare occurrence of multiple shoots as shoot cluster. Studying shoot clusters may offer key information for farmers in enhancing cultivation practices. The very rare occurrence of shoot clusters restricts the chances of effective study. It remains unknown whether shoot cluster is a genetically heritable phenomenon or an intermittent event triggered by environmental factors.

Studies on plant species other than bamboo have found that bud development may involve interactions between hormone and sugar signaling^[12,15−19]^. Diverse microbes live in close association with plants and significantly influence their growth, nutrient uptake, and stress tolerance^[20,21]^. Plants in turn influence the rhizosphere communities by root exudation^[22]^. There is no known correlation between microbes and bud dormancy, while microbial production of plant hormone-like substances may offer some perspectives^[23]^. For example, most rhizosphere microbes produce auxins, which are known to inhibit axillary buds from outgrowth^[24−26]^. Strigolactones and cytokinins are also involved in bud outgrowth and are produced by microbes^[27−30]^. Gibberellic acid is the major plant hormone that breaks bud dormancy^[31]^. Analogs of gibberellic acid, termed gibberellins, are produced by a variety of plant associated microbes^[32−35]^. A microbe strain isolated from the bamboo rhizosphere has recently been demonstrated to produce auxin and promote bamboo growth^[36]^. Bamboo is a perennial plant whose rhizomes coexist with abundant microbes^[37,38]^. The possibility that microbes may have effects on bamboo shoot development remains underexplored. Indeed, as a first step toward this goal, a more complete inventory of the microbial communities associated with the rhizosphere of bamboo shoots and rhizomes is required.

A case of bamboo shoot cluster was encountered, offering us the first chance to study this rare phenomenon. Both bacterial and fungal communities of the rhizome soil microbiome were analyzed to test the working hypothesis that soil microbes may influence shoot cluster formation. This study aims to raise awareness of shoot clusters and contribute toward future research on this topic.

## MATERIALS AND METHODS

### Sample collection and sequencing

Rhizosphere soils of moso bamboo (*Phyllostachys edulis*) were collected at the root areas (about 30–40 cm in depth) with sterile bags on 20 December 2016 (location at 28°34'54.6"N 119°09'59.0"E, Suichang, Zhejiang). Three samples (around 400 g soil) from each rhizome type (samples with 18 shoots and no shoots were from distinct parts within the same rhizome) were collected and stored at +4 °C. Rhizome samples were photographed for documentation of their morphology. However, further sample handling was kept to a minimum in order to prevent contamination, as this material was used for the isolation of microbes. The soil samples were first sieved to exclude roots and coarse gravel, DNA was isolated with the NucleoSpin® Soil kit (Takara, 740780). The isolated DNA was examined by separation in 1.0% agarose and the concentration estimated spectrophotometrically (NanoDrop 2000, ThermoFisher, USA). For bacteria, the V4 16S rRNA region was amplified using the primers 515F 5’-GTGCCAGCMGCCGCGGTAA-3’ and 806R 5’-GGACTACHVGGGTWTCTAAT-3’^[39]^. For fungi, the nuclear rDNA internal transcribed spacer (ITS) region was amplified with primers ITS1F 5’-CTTGGTCATTTAGAGGAAGTAA-3’ and ITS2R 5’-GCTGCGTTCTTCATCGATGC-3’ as previously reported^[40,41]^. The amplified PCR products were sequenced by the Tianjin Novogene Bioinformatics Technology Co., Ltd using paired-end sequencing of the Illumina HiSeq platform.

### Sequence data analysis

Multiplexed raw sequencing data were de-convoluted and quality filtered to remove poor quality reads (average quality score < 25, truncated reads < 50 base pairs, ambiguous bases and frame-shift errors) and potential chimeric sequences with QIIME and Mothur^[42,43]^. Sequences from each library were clustered into operational taxonomic units (OTUs) with 3% differences using the Uparse program^[44]^. A total of 462,263 prokaryotic OTUs and 389,869 fungal OTUs were obtained and classified with ribosomal database project (RDP) classifier^[45]^. The α-diversity indices (Chao, Ace, Shannon) and β-diversity based on both weighted and unweighted Unifrac were calculated and analyzed with the QIIME and Mothur programs. The principle component analysis (PCA) of the samples was performed using QIIME based on the Jaccard distance^[43]^. The linear discriminant analysis effect size method was calculated online (https://huttenhower.sph.harvard.edu/galaxy/) to determine the biomarkers with LDA = 3^[46]^.

### Isolation of *Burkholderia* species

To facilitate root dissection, bamboo root segments of the less lignified sections (5 cm from the root tips) were collected and washed thoroughly. The roots were treated according to the isolation procedures described by White et al., 2015^[47]^. The solution containing microbes was plated on 0.5x PDA medium (SLBT9643, Sigma, USA). Microbial colonies were selected and streaked on a new plate. More than 1,000 single colonies were used in growth-promoting assays with plants. For isolates with significant growth-promoting activity, 16S rDNA was amplified using primers 27F and 1492R, and sequenced. The sequence data of two isolated *Burkholderia* strains is listed in Supplemental Table S4. For the growth-promotion assay, candidate strains were streaked on 0.5x MS medium with rice or bamboo seedlings in aseptic *in vitro* culture without contact with the seedlings. The seedlings were photographed and weighed after one week with or without microbial strains.

### Growth-promotion assay of rice and bamboo seedlings

To test rice and bamboo seedlings *in vitro*, seeds were sterilized with 70% ethanol plus 2% triton X-100 for 3 min, and additionally with 5% sodium hypochlorite for 20 min. The endosperm of sterilized bamboo seeds was excised to eliminate bacterial contamination. Bamboo embryos or intact rice seeds were placed on 0.5x MS medium with 1% sucrose and 1% agar. *Burkholderia* sp. strains YF1 and MY1 were streaked on the medium to avoid direct contact with the sterilized seeds and microbe-free medium was used as control. Strains YF1 and MY1 did not exhibit visible influence on the germination of bamboo embryos*.* One-month-old seedlings were photographed and weighed. For soil-grown seedlings, two inoculation methods were used. One was to inoculate bacteria prior to sowing. Seeds were soaked in bacterial suspensions (OD = 0.6) of strains YF1 or MY1, respectively, for 10 min and then sown in an autoclaved soil mixture of (2:1) peat and vermiculite. The second method applied was to inoculate bacteria after sowing. Bamboo seeds were first sown, then watered three times with bacterial suspensions (OD = 0.6). Growth chamber conditions were 150–200 μmol m^–^^2^ s^–2^, 60% humidity, 12/12 h (light/dark) photoperiod and 23/18 °C (day/night). One-month-old seedlings were dried at 70 °C for 24 h before weight measurement.

### Collection of recorded events of rhizomes with more than ten shoots

In Chinese folklore, events involving the development of clustering shoots within one rhizome are considered as good omens, which are long-cherished and reported in newspapers. In the absence of available scientific documentation, this provided the best possible information resource. These reports were collected and key information, such as time, shoot number, and location were summarized (Supplemental Table S1). The locations of these events were plotted on the map of Zhejiang province, with a color key added using R (version 3.0.3; https://www.rstudio.com/)^[48]^.

### Statistical analysis

Statistical analysis of seedling dry weight was performed with scripts in R (version 3.0.3)^[48]^. Using the nlme package, a linear mixed model with fixed effects for samples, treatments, and their interaction was fitted to the data, plus a random effect for biological repeats as described in Cui et al., 2019^[49]^. The model contrasts were estimated with the multcomp package, and the estimated P-values were subjected to single-step P-value correction. A logarithm of the data was taken before modeling to improve the model fit. The P-values of rice fresh weight were calculated with a two-tailed student's *t*-test using equal variance.

## RESULTS

### Collection of reported events of shoot cluster in China

**Figure 1:**
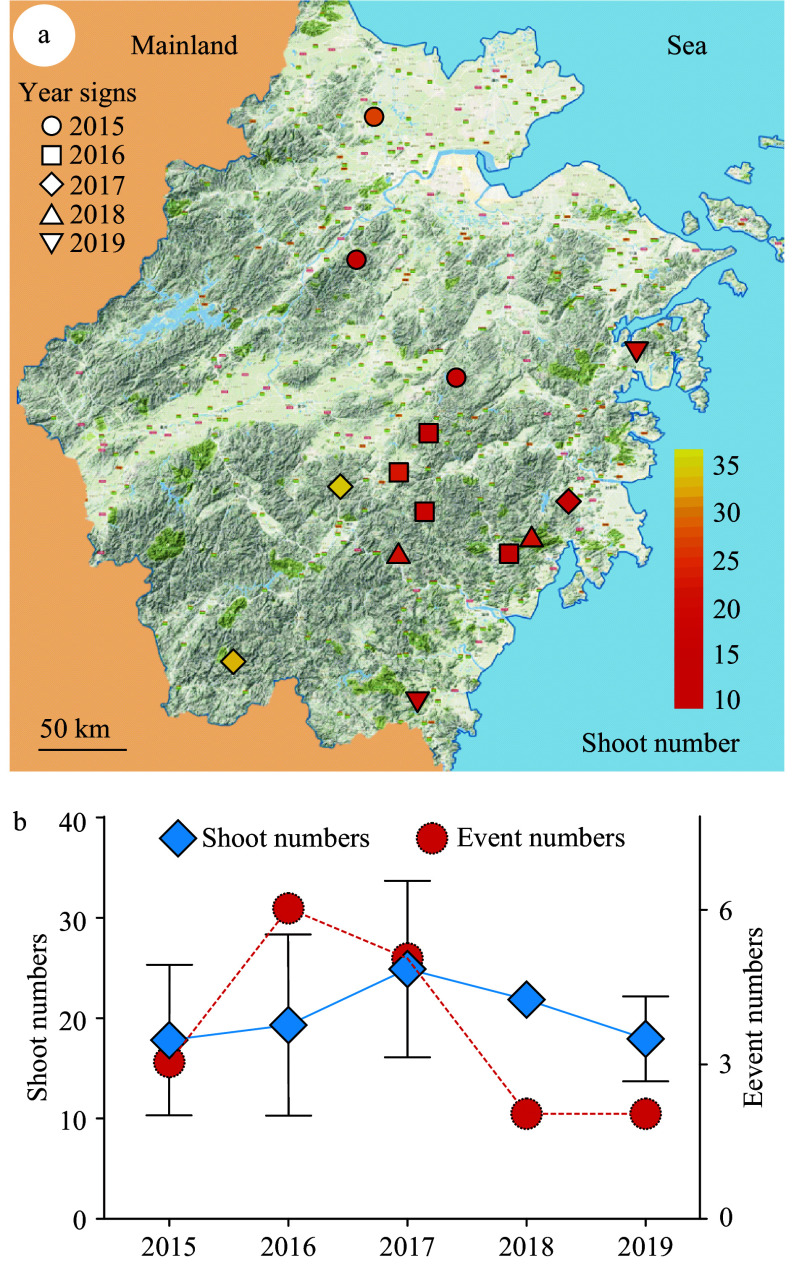
Collection of events of rhizomes with shoot clusters in Zhejiang province. A total of 14 shoot cluster events were recorded. (a) The location of the collected events were marked on the map of Zhejiang province in East China. The recorded events were from 2015–2019, which are represented with different shapes as indicated. The shoot number of each rhizome was from 11–36 as indicated with the color key from red to yellow. Scale bar = 50 km. (b) The number of events recorded each year and the average shoot number of the events for each year were presented. Red circles indicate event number. Blue rhombus indicate yearly average shoot number.

Shoot cluster events are culturally significant and typically reported in the media. Due to their rarity and absence of scientific literature on the subject, newspapers were the only resource available with information regarding shoot clusters. In order to search for clues related to the causes of this rare phenomenon, information from a total of 18 occurrences of shoot clusters that have been reported in Chinese news media were collected. The collected events are from the 2015–2019 from throughout China (Supplemental Table S1). With the exception of four events in two other provinces, all of the events (14 total) were from the Zhejiang province (Supplemental Table S1), one of the regions with most developed bamboo cultivation^[50]^. The locations of these 14 events are indicated on the map of Zhejiang province (Fig. 1a). The numbers of shoots per rhizome and the years of each event were also indicated (Fig. 1a). The timing of events varied: six times in 2016, five in 2017, three in 2015, and only twice each in 2018 and 2019 (Fig. 1b). The average shoot number for all years was similar (Fig. 1b). Importantly, these events were randomly distributed over the entire province with no events occurring twice at the same location (Fig. 1a). This suggests that the phenomenon of shoot cluster was triggered sporadically without the recurrence that would be expected if bamboo genotype had a role. This supports the model that shoot cluster formation is not genetically heritable and prompted exploration of the clustered-shoot rhizome microbiome.

### Location of an 18 shoot rhizome

**Figure 2:**
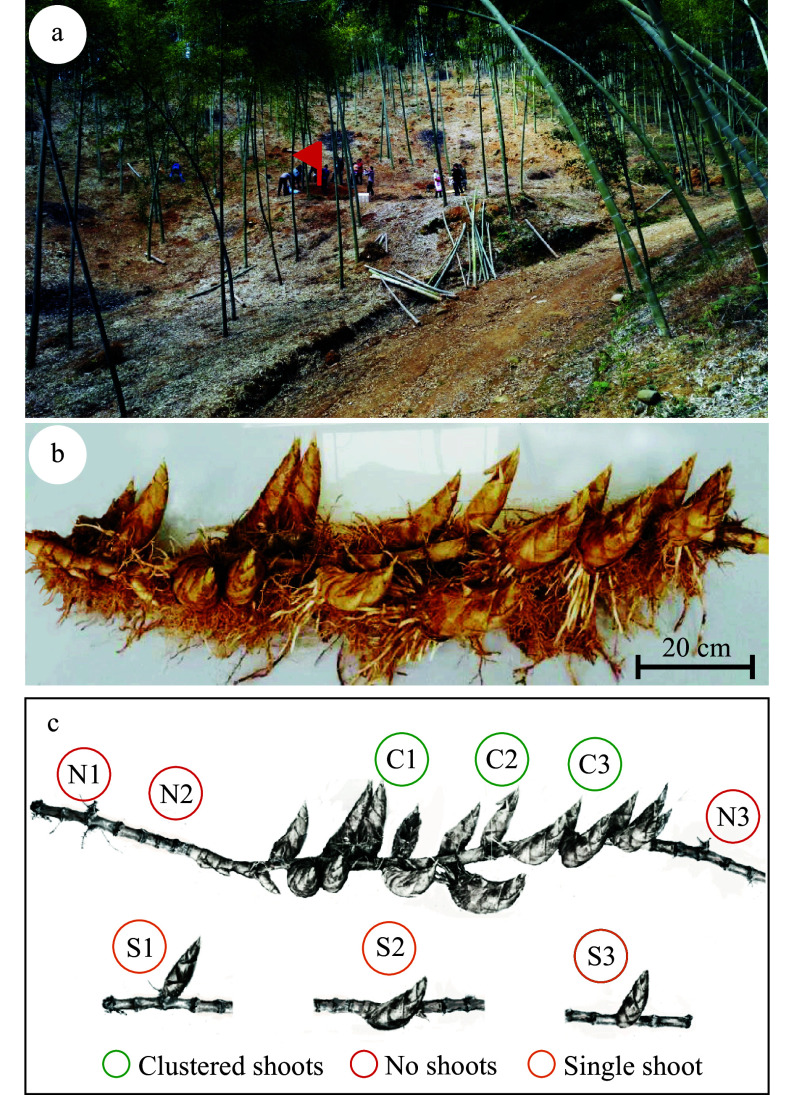
A rhizome with clustered shoots from a moso bamboo plantation. (a) The terrain of the location. The rhizome growing site is indicated with a red flag. (b) Picture of the rhizome with a cluster of 18 shoots. Please note that two shoots are missing from the photograph as they were damaged during harvest. Bar = 20 cm. (c) Schematic diagram of the positions within the rhizome that were sampled for microbial community analysis. Circles with different colors indicate the sampling positions and types.

A shoot cluster was found at a cultivation terrace on a mountain slope facing to the southeast (Fig. 2a). The rhizome growth area was relatively flat. This flat terraced terrain is similar to locations of three other events of shoot cluster according to collected news reports (Supplemental Table S1). The rhizome segment containing the 18 shoot cluster was 1.3 meters long (Fig. 2b), which was sampled out of a single rhizome of ca. 5 m in length. No shoots were found in the regions adjacent to the shoot cluster on both sides, which were each approximately 1.85 m in length. Two shoots were damaged during excavation and 16 remained attached (Fig. 2b). Microbial communities were inventoried using barcode marker library sequencing with DNA isolated from rhizosphere soils. Specifically, samples were collected as follows. Samples C1–C3 were from the rhizosphere surrounding the shoot cluster (Fig. 2c) and each represented approximately 20 cm of length within the clustered rhizome. Samples N1-N3 were from the same rhizome at the left and right of the shoot cluster, with N2 residing ca. 43 cm to the left of the border of the cluster and sample N1 35 cm beyond N2, while sample N3 was 29 cm to the right of the cluster (Fig. 2c). Samples S1–S3 are three single shoot rhizome segments, each derived from different bamboo individuals in the same grove (Fig. 2c). Generally, within the cluster region, rhizome morphology was not significantly changed in terms of thickness or the length between nodes.

### Alpha-diversity of prokaryotic communities

Illumina sequencing of the 16S rRNA amplicon was conducted with DNA isolated and amplified from rhizosphere soil samples (Fig. 2c). A total of 2,972 operational taxonomic units (OTUs) were detected with > 97% identity to bacterial 16S rDNA reference sequences (Supplemental Table S2). All together 1,694 OTUs were detected from the samples of the shoot cluster region, 1,536 OTUs from the single shoot samples, and 1,438 OTUs from the no shoot regions (Supplementary Table S2). This reflected a trend where the higher absolute abundance of prokaryotic OTUs was found in samples with increased shoot number. This trend was also observed in rarefaction curves of the OTUs (Supplemental Fig. S1a). In contrast to bacteria, the number of fungal OTUs did not exhibit this correlation with rhizome shoot number (Supplemental Table S3; Supplemental Fig. S1b). Venn diagrams were constructed with OTUs of the three sample groups. The number of prokaryotic OTUs common to all three samples was 3-10 fold higher than the OTUs unique to each sample (Supplemental Fig. S2a). A similar trend was also observed in the fungal data, where shared OTUs were 1.5-6 fold higher than the unique (Supplemental Fig. S2b). This indicates the microbial communities in the rhizosphere were largely stable between samples.

### Microbial communities of samples with different shoot numbers

**Figure 3:**
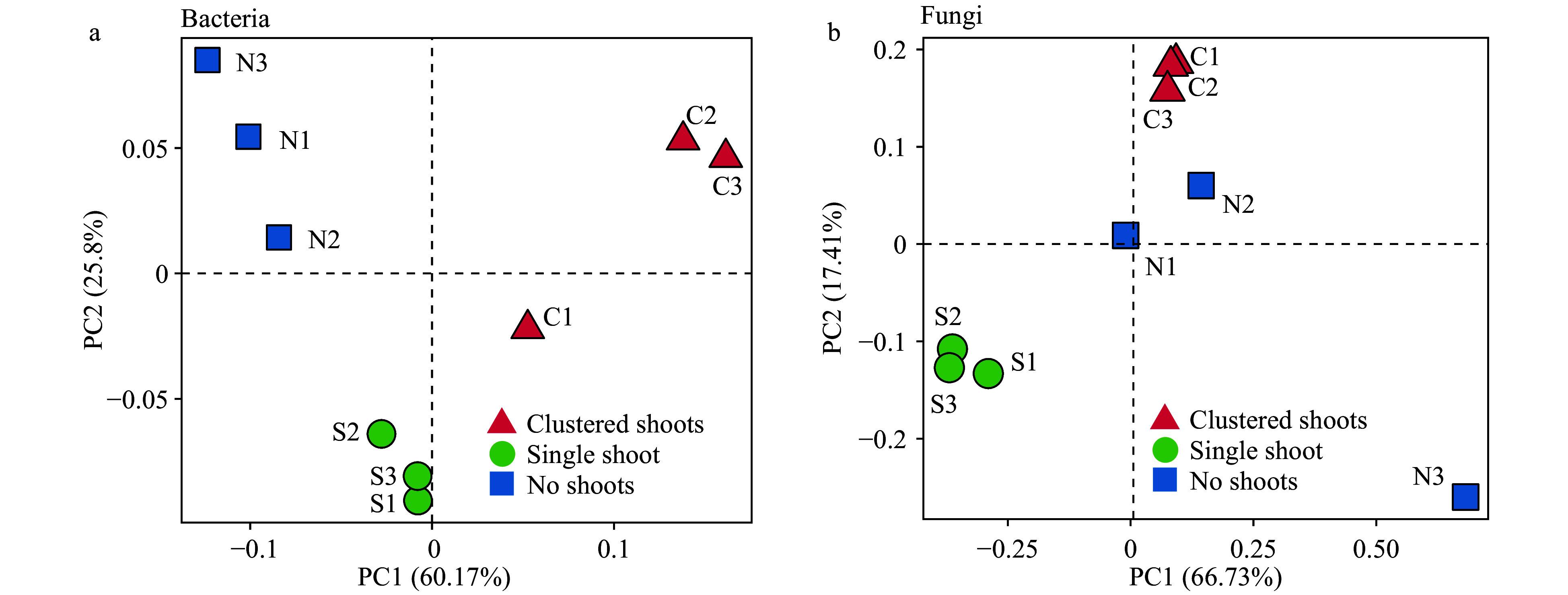
Weighted UniFrac principle component analysis (PCA) of the microbial diversity in three moso bamboo rhizosphere soil sample types. (a) Prokaryotic 16S rDNA gene sequences. Three repeats of each sample type are indicated with red triangles (shoot cluster), green circles (single shoots) and blue square (no shoots). (b) Fungal ITS sequences. Three repeats of each sample type are indicated as in (a).

Principle component analysis (PCA) demonstrated that prokaryote communities clustered according to sample types, however fungal communities did not (Fig. 3a and b). The samples with shoot clusters and no shoots were collected from different segments in the same rhizome. The differentiation of prokaryotes was much more pronounced between these two groups compared to the fungi (Fig. 3a and b).

**Figure 4:**
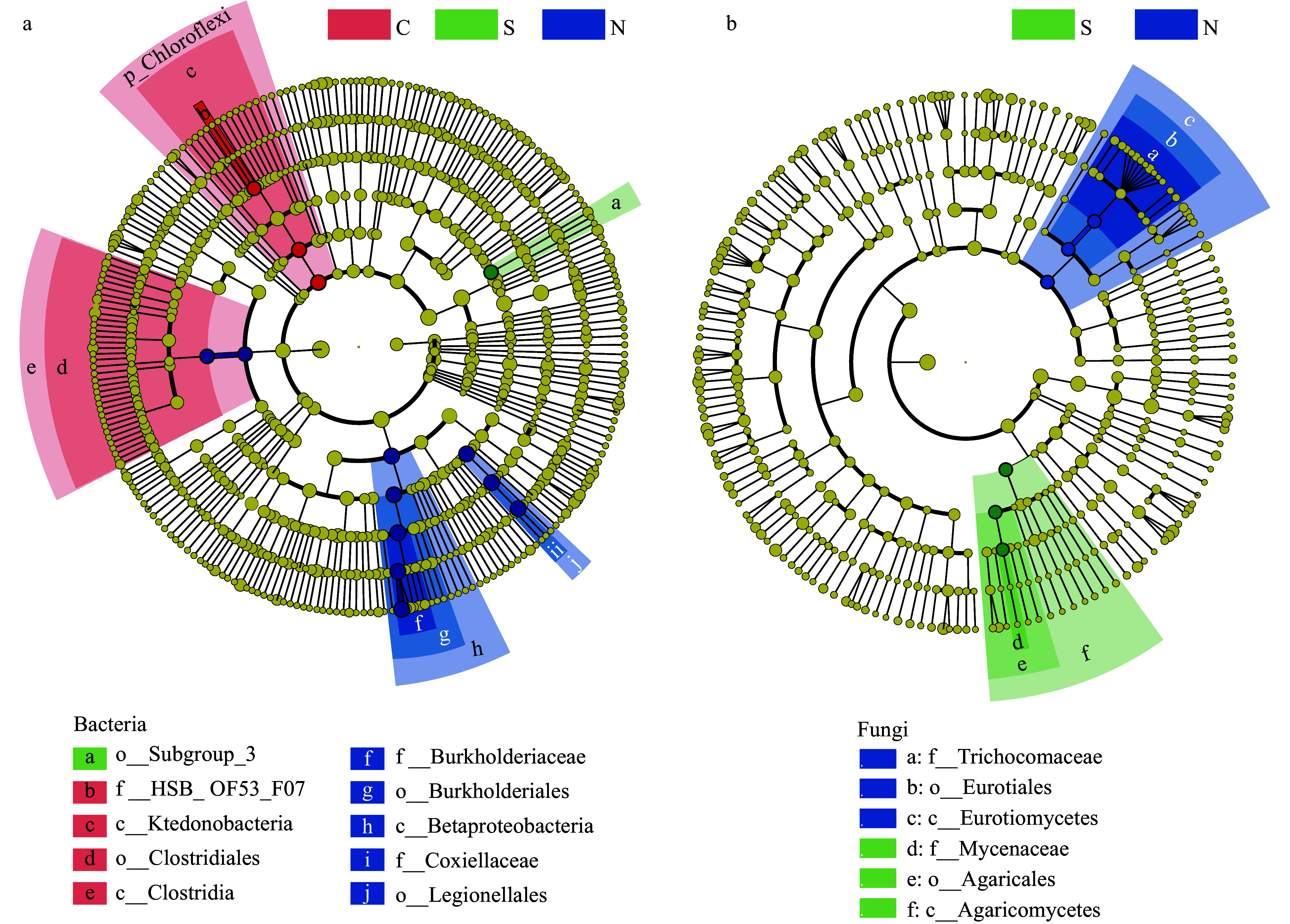
The differential phylogenetic distribution and biomarker microbial taxa of each sample group determined by the linear discriminant analysis (LDA) effect size method. (a) Bacteria. (b) Fungi. LDA scores ≥ 3. Circles indicate phylogenetic levels from phylum to genus. The yellow nodes represent non-significant differential microbes while the nodes in different colors represent the significant differential biomarker microbes. The color sectors indicate sample types as abbreviated: C, clustered shoots, in the pink sector; S, single shoot, in the green sector; N, no shoots, in the blue sector.

To identify the predominant discriminant taxa, the linear discriminant analysis effect size method was employed^[46]^. Representative biomarker taxa were found in each sample group: members of the classes *Clostridia* and *Ktedonobacteria* were significantly enriched in samples with many shoot clusters; the order *Subgroup_3* was the predominant taxa in the single shoot samples, while members of the orders *Burkholderiales* and *Legionellales* were predominant in the no shoot samples (Fig. 4a). In contrast to prokaryotes, fungal biomarker taxa were not identified for the shoot cluster samples (Fig. 4b). Only the fungal classes *Eurotiomycetes* and *Agaricomycetes* were significant in the no shoot and single shoot samples, respectively (Fig. 4b). Taken together, these data suggest that prokaryotic communities may play a more important role in the regulation of rhizome shoot numbers, while fungal communities did not correlate with shoot numbers. Therefore, prokaryotic taxa became the focus of the following studies.

### The most abundant prokaryotic genera in rhizome soil

**Figure 5:**
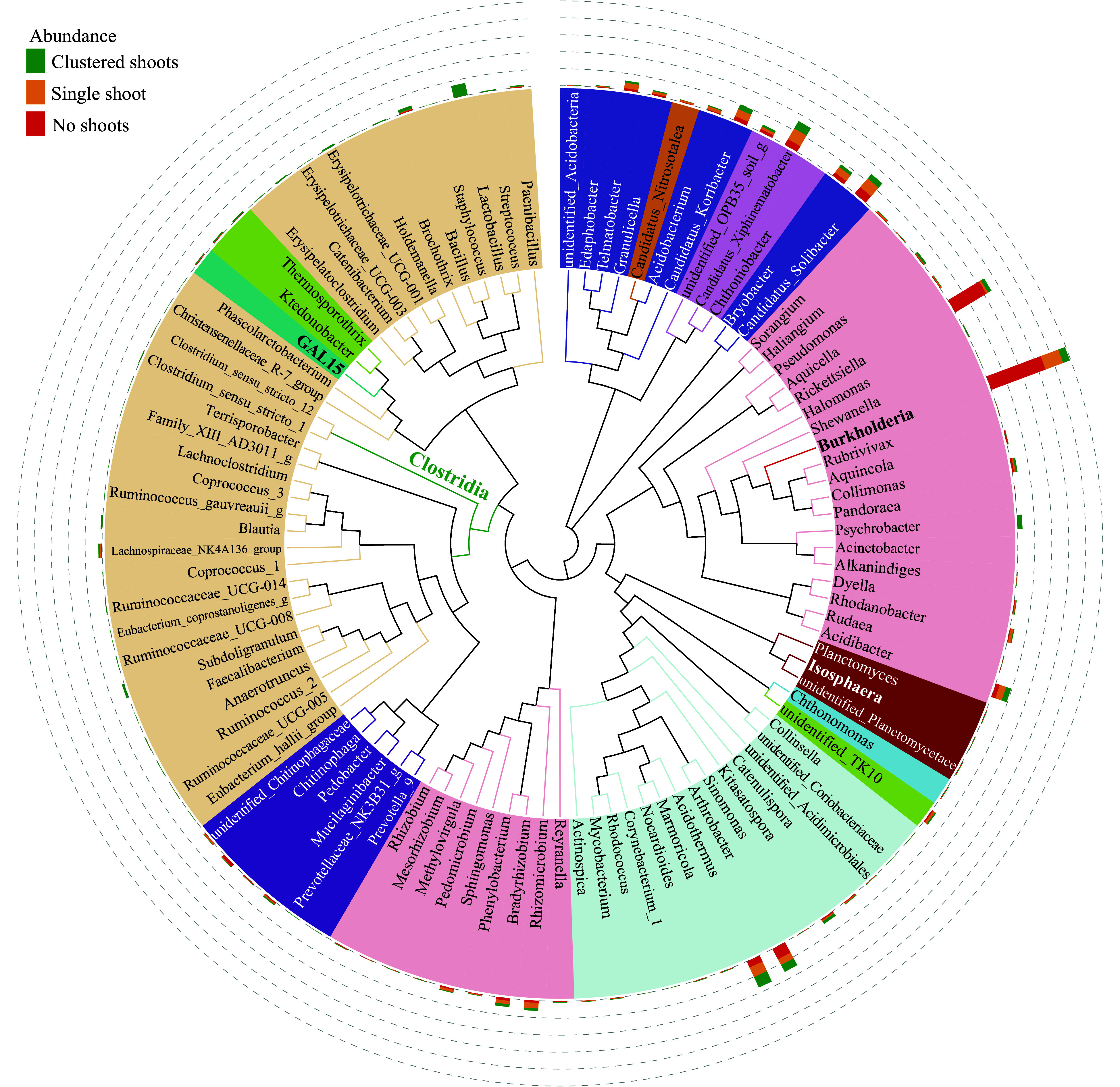
Maximum likelihood phylogeny of the 100 most abundant taxa. Phylogenetic tree based on the most abundant 16S sequences was generated using MEGA 5 with the maximum likelihood method^[77]^. The relative abundance of each genus was proportionally represented with colored bars indicating their sample of origin (green, shoot cluster; orange; single shoots; red, no shoots) and plotted in the outer circles as percent of the most abundant taxa (*Burkholderia*, indicated in bold text).

The 100 most abundant prokaryotic genera were further studied by phylogenetic analysis (Fig. 5), which includes relative abundance. The most abundant genus was *Burkholderia* (Fig. 5), which was also the biomarker of samples with no shoots (Fig. 4a). Interestingly, the abundance of *Burkholderia* was inversely correlated with the number shoots in each sample group; the no shoot sample had the highest abundance, single shoot was next, and then the shoot cluster (Fig. 5; Supplemental Fig. S3). The second most abundant genus *Rickettsiella* (Fig. 5) are primarily intercellular opportunistic pathogens of arthropods, and have a soil-dwelling habit^[51,52]^. Unlike *Burkholderia*, the abundance of *Rickettsiella* exhibited no correlation with shoot number (Fig. 5). Both *Burkholderia* and *Rickettsiella* are proteobacteria, belonging to families of *Burkholderiaceae* and *Coxiellaceae* respectively. These two families were biomarker taxa of the no shoot samples (Fig. 4a). Among the top 100 genera, 22 belonged to the classes *Clostridia* and *Ktedonobacteria*, which were biomarker taxa of the shoot cluster samples (Fig. 5; Fig. 4a).

### Isolates of *Burkholderia* species in growth promotion

**Figure 6:**
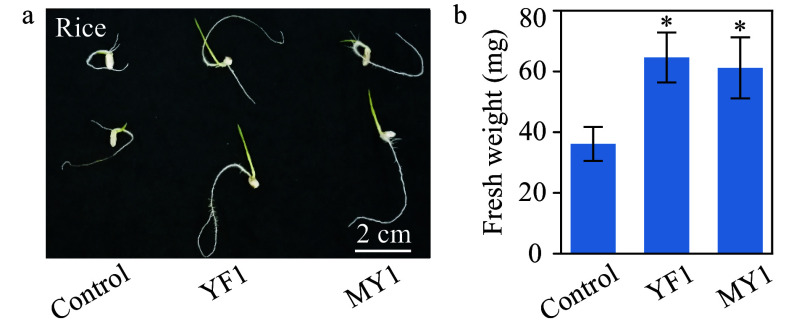
Growth assay of rice seedlings treated with two isolated *Burkholderia* species. (a) Rice seedlings grown *in vitro* with or without *Burkholderia* sp. strain YF1 and MY1. Bar = 2 cm. (b) Quantitative data of fresh weight of rice seedlings treated with strains YF1 and MY1. Error bars represented SD of the means of 20 replicates. This experiment were repeated twice with similar results. * indicates significant groups (*t*-test, *P* < 0.05).

Growth promoting microbes from the rhizosphere of a bamboo shoot cluster were isolated with the aim of obtaining microbes with the ability to modify plant growth. The 16S rRNA sequences of the two isolates (strains YF1 and MY1) exhibited 99% identity to species of *Burkholderia,* placing them in this genus (Supplemental Table S4). These two *Burkholderia* strains were used here in growth assays with two plant species. Significant growth promotion of rice seedlings was detected, as observed visually and quantified in fresh weight (Fig. 6a and b), upon co-cultivation with the two *Burkholderia* strains. This result is consistent with the well-known ability of *Burkholderia* species to promote plant growth^[53]^.

### *Burkholderia* strains tested with bamboo seedlings for growth effects

**Figure 7:**
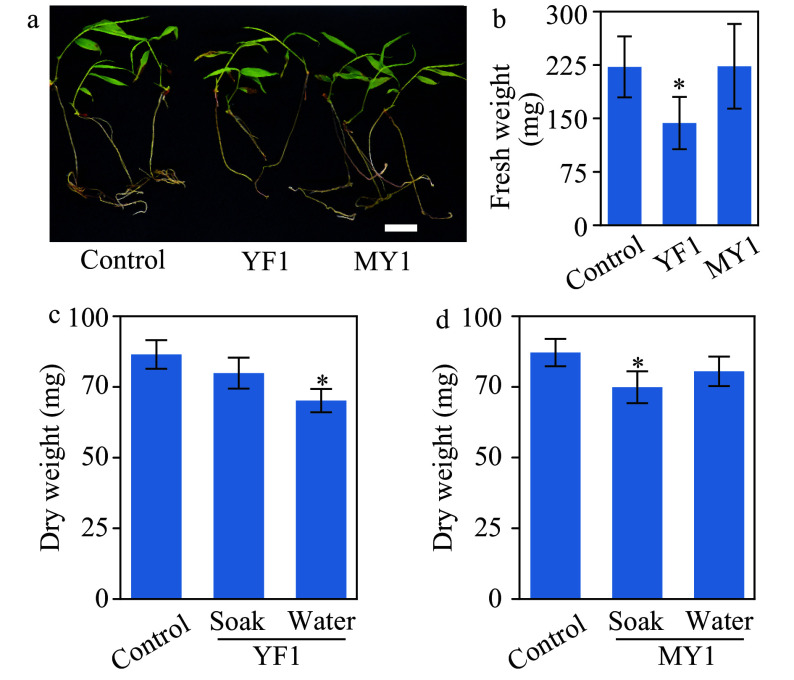
Growth assay of moso bamboo seedlings treated with *Burkholderia* species strains YF1 and MY1. (a) Photographs of one-month-old seedlings with or without application of strain YF1 or MY1. Bar = 2 cm. (b) Quantitative data of fresh weight of the bamboo seedlings in (a). * indicates significant groups (*t*-test, *P* < 0.05). (c, d) Soil-grown bamboo seedlings inoculated with or without strains YF1 or MY1. Inoculation was performed with two methods; soaking bamboo seeds in suspension of strains YF1 or MY1 respectively before sowing (abbreviated as soak), or watering bamboo seedlings with the indicated bacteria suspension three times during growth (abbreviated as water). Data of three repeats were analyzed in a linear mixed model with single-step P-value adjustment. Error bars represent SE of means. * indicates significant groups (*P* < 0.05).

The growth-promoting activities of strains YF1 and MY1 were further tested with *in vitro*-grown bamboo seedlings. Neither strain promoted the growth of bamboo seedlings *in vitro* (Fig. 7a and b). In contrast, strain YF1 diminished bamboo growth in this assay (Fig. 7a and b) compared to control. These results suggested that these two *Burkholderia* strains may have the ability to inhibit bamboo growth. To further confirm this finding, soil-grown seedlings were tested. Two inoculation methods were applied; soaking bamboo seeds in bacterial suspensions prior to sowing, or watering seedlings with a bacteria suspension three times after sowing. Consistent with results from the *in vitro* assay, neither of these two inoculation methods resulted in enhanced growth of bamboo seedlings (Fig. 7c and d). In addition, watering with a strain YF1 suspension and pre-soaking seeds with strain MY1 significantly attenuated bamboo growth (Fig. 7c and d). In summary, the two isolated *Burkholderia* stains had negative effects on the growth of bamboo seedlings.

## DISCUSSION

We have investigated bacterial and fungal community structures in the bamboo rhizosphere soils surrounding varied numbers of shoots. Principle component analysis (PCA) demonstrated that prokaryote communities clustered according to sample types, while fungi did not. Closer study of bacterial communities showed that members of the classes *Clostridia* and *Ktedonobacteria* were significantly enriched in samples with many shoots. These results suggest that bacteria may somehow interact with the bamboo in shoot formation. Members of the species *Burkholderia* were predominant in samples with no shoots and inversely correlated with shoot number. These data represent the first solid scientific investigation of the agriculturally valuable phenomenon, shoot cluster. Due to the limitations presented by scarcity of collected materials and rarity of the phenomenon, firm conclusions cannot be drawn. The possible correlations between these data and shoot cluster are discussed below, and may provide perspectives and insights for further studies.

Bamboo shoots have a special importance in Southern China where fresh shoots are a desired seasonal food that generates much needed income for many farmers especially in rural areas. Multiple shoots are rarely formed from one main root (rhizome). It was fortuitous to have an opportunity to investigate this rare event utilizing microbial ecology tools. Scientific documentation of this phenomenon and collection of primary information will form the basis for hypothesis testing in future studies. The total lack of reports about shoot clusters could be explained by their rarity, but also unpredictability. They are usually discovered by local farmers who lack awareness or interest in the scientific value of the phenomena, not giving researchers notice of the opportunity for further study. Shoot cluster events seem also to have appeared increasingly in recent years according to the literature records (Supplemental Table S1). The extreme cases of shoot clusters collected here may facilitate investigation into typical conditions that are required for the induction of shoot development.

Members of the classes *Clostridia* and *Ktedonobacteria* were prokaryote marker taxa in the microbial communities associated with clustered shoots (Fig. 4a). Among the 100 most abundant genera, 22 belonged to these two classes (Fig. 5)*.* Species in the genus *Clostridium*, belonging to *Clostridia*, have been reported to produce gibberellins, which are essential phytohormones for bud germination^[31,54]^. Members of *Clostridium sensu stricto* are the true representative cluster of *Clostridium*^[55−57]^. *Clostridium sensu stricto* 1 and 12 were among the 100 most abundant genera (Fig. 5). *Ktedonobacteria* is a newly established class, whose members have relatively large genomes, complex metabolism, and aerobic lifestyles^[58]^. Studies on this class are often related to their antibiotic production^[59,60]^. Some bacteria in this class, such as *Streptomyces*, exhibited plant growth promoting activity^[61]^. The *Ktedonobacteria*, have not yet been tested for plant-growth modulating activity. Recently, *Ktedonobacteria* strains were isolated from decayed bamboo stems^[59,62]^, indicating *Ktedonobacteria* genera might influence bamboo via mechanisms distinct from growth-promotion. The effect of bacteria on plant development are usually a combined result of several different species. Elucidation of *Clostridia* and *Ktedonobacteria* function in bamboo bud germination will require significant efforts in bacterial strain isolation and bamboo physiological assays, which are beyond the scope of the current study.

Microbe isolation and screening yielded two *Burkholderia* strains*.* *Burkholderia* was negatively correlated with the number shoots in each sample group. Just as in this study, *Burkholderia species* have been found to be ubiquitous in moso bamboo rhizosphere in bamboo forest in Zhejiang^[63]^. Many *Burkholderia* species have plant-growth promoting activities^[64,65]^ and are known to produce hormone-like substances and enzymes that modulate plant ethylene, auxin, and gibberellin signaling^[65,66]^. The two *Burkholderia* strains isolated here from the bamboo rhizosphere promoted growth in rice (Fig. 6), but attenuated the growth of bamboo seedlings (Fig. 7). This negative effect of *Burkholderia* on bamboo growth is consistent with the result that the abundance of *Burkholderia* were negatively correlated to shoot numbers of rhizomes (Fig. 5 and Supplemental Fig. S3), suggesting a possible function of *Burkholderia* on shoot formation. We do not wish to imply, however, that *Burkholderia* plays the same role in both rhizome shoot formation and seedling development, as they are different processes.

Bamboo shoot initiation assays are the long-term goal of this project. However, bamboo seedlings require at least two years to develop the first rhizome. In light of this, a pre-selection screen was used here to identify microbes with the ability to modify plant growth. Bamboo and rice were selected for screening due to their very different physiologies, which would give deeper insights into microbe plant interactions. The use of rice also has the added benefit of identifying microbes as candidates for development as crop bio-fertilizers. Although both are monocots, bamboo is a terrestrial woody plant with a perennial lifestyle, while rice is herbaceous and a semi-aquatic annual. Annuals and perennials exhibit known differences in their responses to multiple stimuli, such as stress, developmental cues, and hormones^[67−69]^. Thus, it is not unexpected that rice and bamboo exhibit opposite responses to co-cultivation with *Burkholderia* strains. There are other possibilities involving hormone responses for the regulation of plant growth by *Burkholderia*. For example, ethylene is required for bud dormancy release in several plants^[70−72]^. *Bulrkholderia* species produce 1-aminocyclopropane-1-carboxylate (ACC) deaminase, which decreases the ethylene level in plants, via degradation of its direct precursor, ACC^[65]^. The hypothesis would then be that deaminase production could prevent dormancy release in bamboo. The validity of this model will require experimental support in future studies.

Many environmental factors may also be involved in the regulation of bamboo bud germination, as suggested by the shoot cluster reports collected here. The terrain where the shoot cluster frequently occurred is a relatively flat terrace on a slope, which may facilitate deposition of nutrients leached from soils above by rainwater. Rainwater retention in mountainside terraces may also result in hypoxic conditions, which would promote the propagation of anaerobic bacteria. Members of *Clostridia*, the marker taxa for shoot cluster in this study are obligate anaerobes. Temperature might also be a factor. The occurrence of shoot clusters between years varies largely. It peaks in 2016 with six events (Fig. 1b). In China, January 2016 was the coldest winter of the past 30 years, during which numerous plants were killed by a short hard freeze^[73]^. Freezing exposure has been reported to release bud dormancy in multiple perennial trees^[74−76]^. Thus, it is plausible that the short exposure to freezing temperatures in 2016 could have contributed to shoot cluster occurrence. However, environmental factors would influence areas on a large scale, while reported shoot cluster events have remained sporadic and isolated. Nonetheless, a role for environmental factors cannot be excluded and further exploration of both biotic and abiotic factors, or possible interactions between them, are warranted.

In conclusion, this is the first study on the rare phenomenon of shoot clusters. We analyzed the soil microbial community, recorded the terrain of the locations where it occurs, and discussed the influence of environmental factors. This information provides a reference point for future studies of this understudied topic.

## SUPPLEMENTARY DATA

Supplementary data to this article can be found online.
