# Supplementary material for: Case study of a rhizosphere microbiome assay on a bamboo rhizome with excessive shoots
Source: For Res (Fayettev). 2021 Jun 24;1:10. doi: 10.48130/FR-2021-0010 (PMC11524271; doi:10.48130/FR-2021-0010)
Supplement: Supplementary file 1 — Supplementary data to this article can be found online. [file FR-2021-0010-S1.zip › 10.48130_FR-2021-0010-Suppl-FigureS3.pdf]

Supplementary Figure 3

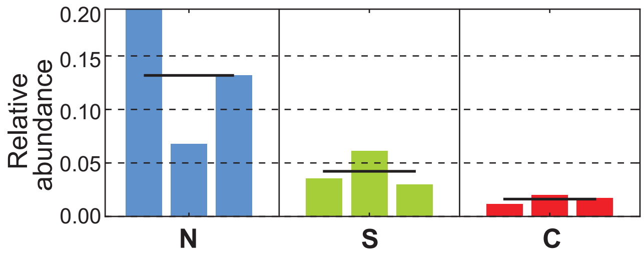

Supplementary Fig. 3. Relative abundance of *Burkholderia* bacteria in each replicates. The abundance in each replicate of the most abundant genera *Burkholderia* in Fig. 5 are shown individually. The bar in each group represents the average abundance of the three samples. Abbreviations: N, no shoots; S, a single shoot; C, clustered shoots.
