# Supplementary material for: Case study of a rhizosphere microbiome assay on a bamboo rhizome with excessive shoots
Source: For Res (Fayettev). 2021 Jun 24;1:10. doi: 10.48130/FR-2021-0010 (PMC11524271; doi:10.48130/FR-2021-0010)
Supplement: Supplementary file 1 — Supplementary data to this article can be found online. [file FR-2021-0010-S1.zip › 10.48130_FR-2021-0010-Suppl-FigureS1.pdf]

Supplementary Fig. 1

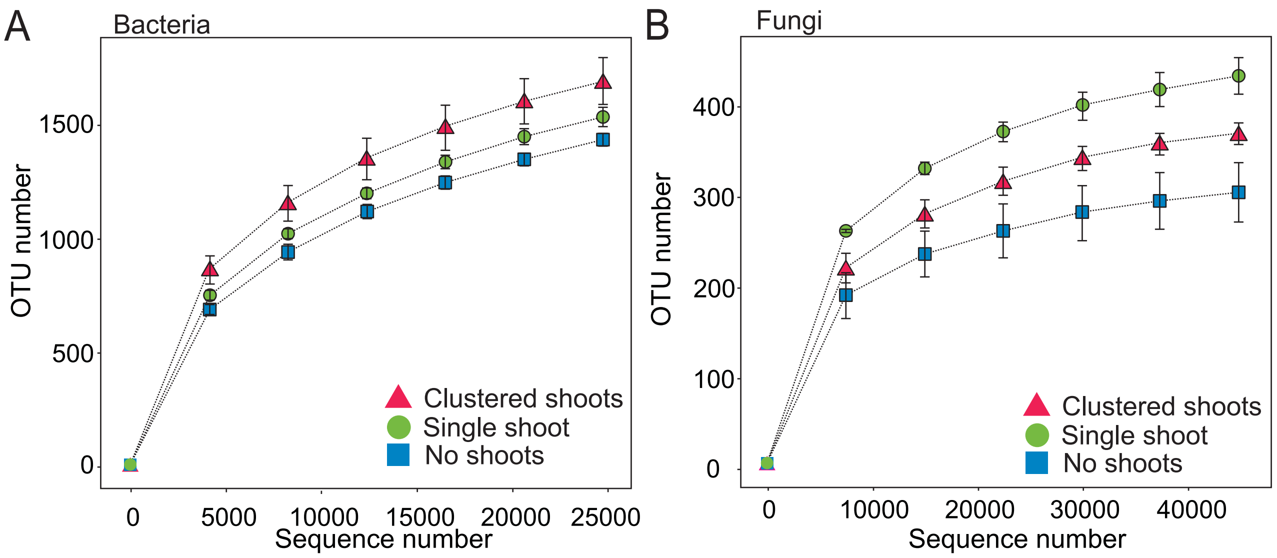

Supplementary Fig. 1. Rarefaction curves of OTUs at 97% similarity for samples from clustered moso bamboo shoots, a single shoot, and no shoots. (A) Bacteria. (B) Fungi. Samples from clustered shoots, a single shoot, and no shoots are indicated by red triangle, green circle, and blue square respectively. The average OTUs number of three samples of each group are shown.
